# Cardiovascular disease risk profiles in inflammatory joint disease entities

**DOI:** 10.1186/s13075-017-1358-1

**Published:** 2017-07-03

**Authors:** Grunde Wibetoe, Eirik Ikdahl, Silvia Rollefstad, Inge C. Olsen, Kjetil Bergsmark, Tore K. Kvien, Anne Salberg, Dag Magnar Soldal, Gunnstein Bakland, Åse Lexberg, Bjørg-Tilde Fevang, Hans Christian Gulseth, Glenn Haugeberg, Anne Grete Semb

**Affiliations:** 1 Preventive Cardio-Rheuma Clinic, Department of Rheumatology, Diakonhjemmet Hospital, PO Box 23, Vinderen, N-0319, Oslo, Norway; 2 Department of Rheumatology, Diakonhjemmet Hospital, Oslo, Norway; 3 Lillehammer Hospital for Rheumatic Diseases, Lillehammer, Norway; 4 Department of Rheumatology, Hospital of Southern Norway, Kristiansand, Norway; 5 Department of Rheumatology, University Hospital of Northern Norway, Tromsø, Norway; 6 Department of Rheumatology, Vestre Viken Hospital, Drammen, Norway; 7 Department of Rheumatology, Haukeland University Hospital, Bergen, Norway; 8 Department of Rheumatology, Betanien Hospital, Skien, Norway; 9 Department of Rheumatology, Martina Hansens Hospital, Bærum, Norway; 10 Faculty of Medicine, University of Oslo, Oslo, Norway

**Keywords:** Rheumatoid arthritis, Spondyloarthropathies, Spondyloarthritis, Cardiovascular, Epidemiology

## Abstract

**Background:**

Patients with inflammatory joint diseases (IJD) have increased risk of cardiovascular disease (CVD). Our aim was to compare CVD risk profiles in patients with IJD, including rheumatoid arthritis (RA), axial spondyloarthritis (axSpA) and psoriatic arthritis (PsA) and evaluate the future risk of CVD.

**Methods:**

The prevalence and numbers of major CVD risk factors (CVD-RFs) (hypertension, elevated cholesterol, obesity, smoking, and diabetes mellitus) were estimated in patients with RA, axSpA and PsA. Relative and absolute risk of CVD according to Systematic Coronary Risk Evaluation (SCORE) was calculated.

**Results:**

In total, 3791 patients were included. CVD was present in 274 patients (7.2%). Of those without established CVD; hypertension and elevated cholesterol were the most frequent CVD-RFs, occurring in 49.8% and 32.8% of patients. Patients with PsA were more often hypertensive and obese. Overall, 73.6% of patients had a minimum of one CVD-RF, which increased from 53.2% among patients aged 30 to <45 years, to 86.2% of patients aged 60 to ≤80 years. Most patients (93.5%) had low/moderate estimated risk of CVD according to SCORE. According to relative risk estimations, 35.2% and 24.7% of patients had two or three times risk or higher, respectively, compared to individuals with no CVD-RFs.

**Conclusions:**

In this nationwide Norwegian project, we have shown for the first time that prevalence and numbers of CVD-RFs were relatively comparable across the three major IJD entities. Furthermore, estimated absolute CVD risk was low, but the relative risk of CVD was markedly high in patients with IJD. Our findings indicate the need for CVD risk assessment in all patients with IJD.

**Electronic supplementary material:**

The online version of this article (doi:10.1186/s13075-017-1358-1) contains supplementary material, which is available to authorized users.

## Background

Patients with inflammatory joint diseases (IJD) have an increased risk of atherosclerotic cardiovascular disease (CVD) [1–3]. Today, RA is recognized by the European Society of Cardiology (ESC) as a risk factor for CVD [4]. Furthermore, the European League Against Rheumatism (EULAR) recommendations for CVD risk management advocate that CVD risk assessments should be undertaken every 5 years for patients with rheumatoid arthritis (RA), ankylosing spondylitis (AS) and psoriatic arthritis (PsA) [5]. In the Norwegian Collaboration on Atherosclerotic disease in patients with Rheumatic joint diseases (NOCAR) project, systematic CVD risk assessment has been implemented into clinical practice in rheumatology outpatient clinics [6].

The aforementioned EULAR recommendations underline the importance of management of conventional CVD risk factors (CVD-RFs), in addition to control of rheumatic disease activity in order to decrease the risk of atherosclerotic CVD [5]. In the general population, five major CVD-RFs; hypertension (HT), elevated total cholesterol (TC), smoking, obesity and diabetes mellitus account for 80% of myocardial infarction (MI) [7] and half of cardiovascular mortality [8]. Moreover a recent meta-analysis found that the five major CVD-RFs also increased the risk of CVD in patients with RA [9].

Externally validated CVD risk algorithms specifically developed for IJD patients are currently lacking. Thus, the use of CVD risk calculators developed and validated for the general population are currently the most reasonable approach in patients with IJD. Systematic Coronary Risk Evaluation (SCORE) estimates the 10-year risk of a fatal atherosclerotic CVD event [10]. Since the SCORE algorithm appears to underestimate the CVD risk in IJD patients [11, 12], the 2016 updated EULAR recommendations advise applying a 1.5 multiplication factor when estimating CVD risk in patients with RA [5]. In contrast to SCORE, which estimates the absolute risk of a future CVD event, relative risk describes the risk in a patient compared to individuals with no CVD-RFs (e.g. a relative risk of two implies that the individual has two-times the absolute risk of CVD).

Studies comparing the prevalence of CVD-RFs and estimated CVD risk across different IJD entities are limited [13, 14]. Despite awareness of frequent CVD comorbidities in IJD patients, time constraints in the rheumatic outpatient clinics may represent a major obstacle to the management of CVD-RFs. Thus, efficient and swift CVD risk assessment programs may facilitate the undertaking of CVD risk evaluation in rheumatology outpatient clinics in this high-risk patient population. We aimed to evaluate whether there are differences in the CVD risk profiles between patients with RA, axSpA or PsA. To accomplish this, our objective was to estimate and compare the prevalence and quantities of major CVD-RFs and estimated CVD risk according to the SCORE algorithm and the relative risk across different IJD entities and age strata.

## Methods

In the nationwide NOCAR project, annual CVD risk assessment is implemented in the rheumatology outpatient clinics according to previous EULAR recommendations [15]. The NOCAR project has been approved by Data Protection Officers (ref 2014/11741) and being a quality assurance project, informed consent and ethics board approval was not required. All patients with IJD who were between 30 and 80 years of age were eligible for inclusion in the project. The CVD risk assessment includes self-reported CVD-RFs (diabetes mellitus, smoking and anthropometric measures for calculation of body mass index (BMI)), use of CVD preventive medication and presence of CVD comorbidities. In addition, lipids (total cholesterol (TC), low-density lipoprotein cholesterol (LDL-c), high-density lipoprotein cholesterol (HDL-)] and triglyceride (TG)) are added to the routine rheumatology laboratory tests, and blood pressure (BP) measurements are undertaken by rheumatology nurses at the time of the clinical joint examination. In the case of initial elevated BP (i.e. >140/90 mmHg), health personnel are instructed to conduct three consecutive measurements and record the average of the two last measurements. Furthermore, risk of fatal CVD events in the coming 10 years is estimated by the SCORE algorithm.

In these analyses, we included patients with RA, axial spondyloarthritis (axSpA) (e.g. nonradiographic axSpA and AS) or PsA, who at minimum had recordings of BP and/or lipid levels. Currently, NOCAR data have been recorded in seven clinics located across Norway (Oslo (Diakonhjemmet Hospital), Lillehammer (Hospital for Rheumatic Diseases), Kristiansand (Hospital of Southern Norway), Skien (Betanien Hospital), Bergen (Haukeland University Hospital), Drammen (Vestre Viken Hospital) and Tromsø (University Hospital of Norther Norway)).

CVD-related variables were included, in addition to demographic, socioeconomic, and rheumatic-disease-related variables and antirheumatic medication (Table 1). In the current project, axSpA included both radiographic (AS) and nonradiographic axSpA.

**Table 1:** Patient characteristics

Variables	IJD (n = 3517)	RA (n = 1961)	axSpA (n = 835)	PsA (n = 721)	*p*
Female, *n* (%)	2046 (58.2)	1389 (70.8)	293 (35.1)	364 (50.5)	<0.001
Age in years, mean ± SD	55.1 ± 11.6	59.1 ± 11.2	48.4 ± 9.6	52.0 ± 10.0	<0.001
Age 30 to <45 years, *n* (%)	774 (22.0)	245 (12.4)	338 (40.5)	191 (26.5)	-
Age 45 to <60 years, *n* (%)	1485 (42.2)	729 (37.2)	385 (46.1)	371 (51.5)	-
Age 60 to ≤80 years, *n* (%)	1258 (35.8)	987 (50.3)	112 (13.4)	159 (22.1)	-
Working/student, *n* (%)	1755 (52.4)	806 (43.0)	543 (68.4)	406 (59.9)	<0.001
Education in years, mean ± SD	12.9 ± 3.3	12.4 ± 3.3	13.8 ± 3.1	13.3 ± 3.0	<0.001
Conventional CVD risk factors					
Total cholesterol (mmol/l), mean ± SD	5.37 ± 1.1	5.38 ± 1.09	5.29 ± 1.05	5.41 ± 1.07	0.065
Triglycerides (mmol/l), median (IQR)	1.30 (0.94, 1.86)	1.24 (0.93, 1.74)	1.34 (0.91, 1.92)	1.43 (1.00, 2.16)	<0.001
LDL-c (mmol/l), mean ± SD	3.29 ± 0.97	3.27 ± 0.97	3.29 ± 0.96	3.37 ± 0.97	0.056
HDL-c (mmol/l), mean ± SD	1.56 ± 0.51	1.64 ± 0.52	1.47 ± 0.48	1.45 ± 0.46	<0.001
Body mass index (kg/m^2^), mean ± SD	26.5 ± 4.6	26.1 ± 4.5	26.4 ± 4.3	28.0 ± 4.9	<0.001
Systolic BP (mmHg), mean ± SD	132.0 ± 17.1	132.1 ± 17.3	129.8 ± 16.7	133.9 ± 16.9	<0.001
Diastolic BP (mmHg), mean ± SD	80.7 ± 9.5	80.0 ± 9.2	80.9 ± 10.1	82.4 ± 9.4	<0.001
Rheumatic disease related variables					
RF IgM+, *n* (%)	-	787 (66.5)	-	-	-
ACPA+, *n* (%)	-	1066 (77.3)	-	-	-
HLA-B27+, *n* (%)	-	-	445 (85.6)	-	-
Disease duration in years, median (IQR)	8.4 (3.8, 16.6)	8.3 (4.0, 15.4)	10.3 (3.8, 20.8)	7.6 (3.1, 15.3)	<0.001
ESR (mm/h), median (IQR)	9 (5, 17)	10 (5, 19)	7 (3, 15)	8 (4, 16)	0.095
CRP (mg/l), median (IQR)	3 (1, 5)	3 (1, 6)	3 (1, 5)	3 (1, 5)	<0.001
DAS28 (ESR), mean ± SD	-	2.59 ± 1.24	-	2.51 ± 1.28	-
Remission (<2.6), *n* (%)	-	912 (55.2)	-	313 (55.9)	-
Low disease activity (2.6 to <3.2), *n* (%)	-	273 (16.5)	-	90 (16.1)	-
Moderate disease activity (3.2 to ≤5.1), n (%)	-	405 (24.5)	-	137 (24.5)	-
High disease activity (>5.1), n (%)	-	63 (3.8)	-	20 (3.6)	-
ASDAS (CRP), median (IQR)	-	-	1.59 (1.04, 2.53)	1.54 (0.99, 2.31)	-
Inactive (<1.3), *n* (%)	-	-	236 (37.8)	182 (40.4)	-
Moderate (1.3 to <2.1), *n* (%)	-	-	165 (26.4)	129 (28.6)	-
High (2.1 to ≤3.5), *n* (%)	-	-	184 (29.4)	112 (24.8)	-
Very high (>3.5), *n* (%)	-	-	40 (6.4)	28 (6.2)	-
BASDAI, median (IQR)	-	-	1.29 (0.25, 4.13)	1.09 (0.26, 3.73)	-
Antirheumatic medication, current use					
Glucocorticoids, *n* (%)	641 (18.2)	575 (29.3)	17 (2.0)	49 (6.8)	<0.001
Methotrexate, *n* (%)	1451 (41.3)	1100 (56.1)	54 (6.5)	297 (41.2)	<0.001
Other sDMARDs, *n* (%)	1925 (54.7)	1462 (74.6)	88 (10.5)	375 (52.0)	<0.001
bDMARDs, *n* (%)	1750 (49.8)	848 (43.2)	518 (62.0)	384 (53.3)	<0.001

*ACPA* anti-cyclic citrullinated peptide antibodies, *ASDAS*-*CRP* Ankylosing Spondylitis Disease Activity SCORE using CRP, *axSpA* axial spondyloarthtis, *BASDAI* Bath ankylosing spondylitis disease activity index, *BP* blood pressure, *BMI* body mass index, *CVD* cardiovascular disease, *CRP* C-reactive protein, *DAS28*-*ESR* Disease Activity Score using 28 joint-ESR, *ESR* erythrocyte sedimentation rate, *HDL*-*c* high-density lipoprotein-cholesterol, *HLA*-*B27* histocompatibility antigen HLA-B27, *IJD* inflammatory joint diseases, *LDL*-*c* low-density lipoprotein-cholesterol, *PsA* Psoriatic arthritis, *RA* rheumatoid arthritis, *RF* rheumatoid factor, *sDMARDs* and *bDMARDs* synthetic and biologic disease-modifying antirheumatic drugs

Established atherosclerotic CVD was defined by self-reported MI, percutaneous coronary intervention (PCI) and/or coronary artery bypass graft (CABG) surgery, peripheral arterial disease (PAD), stroke and/or transient ischemic attack (TIA). In the absence of CVD and/or no reported CVD, patients were classified as being without established CVD. The prevalence of conventional CVD-RFs was assessed in all individuals without documented CVD. HT was defined as presence of self-reported HT, current use of antihypertensive treatment (AntiHT) or systolic BP (sBP)/diastolic BP (dBP) ≥140/90 mmHg [16]. In line with the acknowledged National Health and Nutrition Examination Survey (NHANES), elevated TC was defined as TC ≥6.2 mmol/l (240 mg/dl) [17] and/or use of lipid-lowering therapy (LLT) [8]. Body mass index (BMI) ≥30 kg/m^2^ was classified as obesity. Current smoking and diabetes mellitus were defined by self-reported presence of these CVD-RFs.

The CVD risk (10-year risk of a fatal CVD event) was estimated according to the electronic updated SCORE algorithm for countries with low risk of CVD, available online at HeartScore (www.HeartScore.org), which includes HDL-c. Patients were eligible for CVD risk assessment by SCORE in the absence of diabetes mellitus, established CVD and use of LLT and/or AntiHT. In the case of incomplete reporting of diabetes mellitus and/or status of LLT/AntiHT use, patients were analyzed as non-diabetic and/or non-users of LLT/AntiHT treatment, respectively. In RA, the SCORE calculations were performed with and without applying the EULAR 1.5 multiplication factor [5]. Relative risk was calculated in all patients eligible for assessment of CVD risk using the SCORE algorithm, by finding the nearest sBP value from 120 to 180 mmHg with 20 mmHg increments and the nearest TC integer from 4 to 8 mmol/l for smokers and non-smokers, as published in the latest ESC guidelines [4]. Depending on the estimated relative risk (RR), patients were subsequently grouped into no increased risk (RR = 1), moderately increased risk (RR = 2) or highly increased risk of future CVD (RR = 3–12).

Sensitivity analyses were conducted to compare available cases (all included patients) and complete cases (excluding patients with missing or incomplete recorded CVD comorbidities) in: (1) the prevalence of specific CVD-RFs, (2) the quantity of CVD-RFs and (3) the estimated 10-year risk of CVD events. In further sensitivity analyses, we compared the percentage of patients with a particular CVD-RF among all patients included (percentage) to the percentage of patients included with valid observations of a CVD-RF (valid percentage).

### Statistical analyses

Estimations of CVD-RF prevalence were conducted after imputing missing data as absence of the relevant CVD-RF. Furthermore, these estimations were then compared to estimates of CVD-RFs among only valid observations. Likewise, the quantity of CVD-RFs was estimated in patients without established CVD, again using a conservative approach and imputing missing data as absence of the relevant CVD-RFs. To compare CVD risk profiles across IJD entities in patients with similar age, patients were grouped into the age intervals 30 to <45, 45 to <60 and 60 to ≤80 years. These age groups were chosen arbitrarily before conducting any analyses to provide sufficient sample size for reliable estimations. Additional analyses were performed to evaluate the association between CVD-RFs and rheumatic disease activity, rheumatoid factor (RF)/anti-citrullinated protein antibodies (ACPA) or current use of antirheumatic medication. Correlation analysis using the Pearson partial correlation method was also conducted to evaluate the association between rheumatic disease activity and levels of total cholesterol. Group differences were evaluated using the chi-square test for dichotomous endpoints. Fisher’s exact test was applied in cases of low cell counts. For continuous dependent variables, one-way analysis of variance (ANOVA) was conducted, whereas Welch ANOVA was used if homogeneity of variance was violated. Furthermore, the Mann-Whitney U test or Kruskal-Wallis H test was used for continuous variables with non-normal distributions. Statistical significance was set at *p* ≤ 0.05 and all statistical analyses were performed using the SPSS Statistics for Windows, Version 21.0 [18].

## Results

In total, 3791 patients fulfilled the inclusion criteria. Established CVD was reported in 274 individuals (7.2%), leaving 3517 patients (RA, n = 1961; axSpA, n = 835; axSpA and PsA, n = 721) without documented CVD (including 727 individuals with missing or incomplete reporting of atherosclerotic CVD). The characteristics of patients with RA, axSpA and PsA were as follows. First, patients with RA were older (*p* < 0.001) and more often female (*p* < 0.001) compared to patients with axSpA and PsA. Second, patients with axSpA had slightly longer median disease duration than those with PsA. Third, while use of glucocorticoids, methotrexate and other synthetic disease-modifying antirheumatic drugs (sDMARDs) were more common in RA, treatment with biologic disease-modifying antirheumatic drugs (bDMARDs) was more frequent in axSpA. Overall, the patients with IJD who were included in this project had quite low disease activity in terms of both biochemical inflammatory markers and composite disease activity scores (Table 1).

**Fig. 1:**
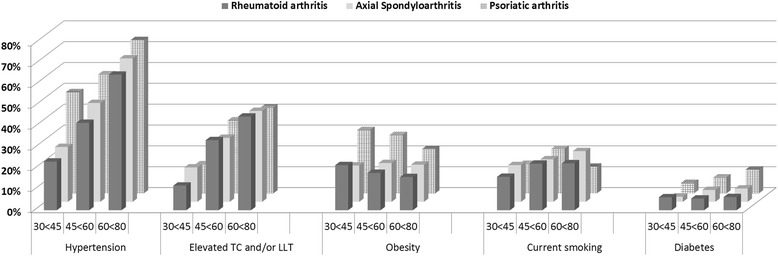
Prevalence of hypertension, elevated total cholesterol (*TC*), obesity, current smoking and diabetes mellitus in patients with different inflammatory joint diseases and age strata (30 to <45 years, 45 to <60 years and 60 to ≤80 years), without established atherosclerotic cardiovascular disease (CVD). Hypertension was defined as the presence of self-reported hypertension, use of antihypertensive treatment and/or systolic BP/diastolic BP >140/90. Elevated TC was defined as TC >6.2 and/or use of lipid-lowering therapy. Body mass index >30 kg/m^2^ was classified as obesity, whereas current smoking and diabetes mellitus was defined by the self-reported presence of these CVD risk factors

The prevalence of conventional CVD-RFs is shown in Fig. 1. HT was the most frequent CVD-RF and was present in 49.8% of all patients with IJD. The rate of HT increased with age and ranged from 28.0% to 66.4% in the youngest versus oldest age stratum. Patients with PsA were most often hypertensive compared to patients with RA and axSpA.

TC was elevated in 32.8% of all patients with IJD. TC also increased with age, ranging from 14.2 to 43.9%, in the youngest and the oldest age stratum, respectively. Overall, the frequency of elevated TC did not differ significantly between IJD entities within similar age intervals.

Obesity was present in 17.4% of IJD patients. In contrast to other CVD-RFs, the frequency of obesity did not increase with advancing age. Obesity was significantly more prevalent among patients with PsA than the other IJD entities.

Current smoking was reported by 20.1% of patients, with no significant differences between the IJD entities apart from fewer smokers among older patients with PsA. Last, diabetes mellitus was reported in 4.8%. Despite borderline significantly higher frequency of diabetes mellitus among younger patients with RA, there were no marked differences between the IJD entities.

Patients with RA and axSpA with high or very high rheumatic disease activity had higher frequency of hypertension than those with low disease activity or remission (see Additional file 1: Table S7). Patients with AxSpA with high disease activity were also more often current smokers. However, patients with high/very high rheumatic disease activity were older. There was no association between total cholesterol level and rheumatic disease activity when adjusting for age and sex (data not shown). Stratification by RF and ACPA status revealed that RF+ and ACPA+ patients with RA were more often current smokers (see Additional file 1: Table S8). The prevalence of CVD-RFs in patients with IJD according to current use of bDMARDs is presented in supplementary files (see Additional file 1: Table S9).

In sensitivity analyses, the prevalence of CVD-RFs was comparable when comparing available cases and complete cases in which complete CVD comorbidity had been recorded (data not shown). Furthermore, the percentage of all patients included who had a particular CVD-RF (percentage) was comparable to the percentage of patients included who had only valid observations (valid percent) of the specific CVD-RF (see Additional file 1: Table S1). This finding was consistent across the three IJD entities and all age categories.

**Fig. 2:**
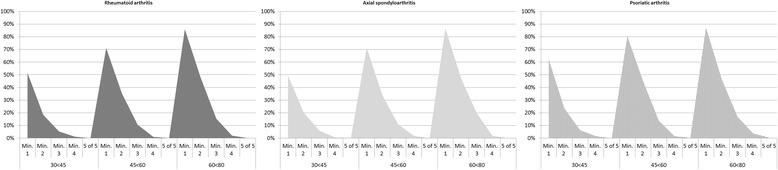
Percentage of patients with rheumatoid arthritis, axial spondyloarthritis or psoriatic arthritis, aged 30 to <45 years, 45 to <60 years and 60 to <80 years with a minimum (*min*.) of 1, 2, 3, 4 and 5 out of 5 conventional cardiovascular risk factors

In the three strata of increasing age, 53.2%, 73.5% and 86.2% of patients with IJD had a minimum of one CVD-RF, respectively (see Additional file 1: Table S2). The frequency of patients with at least one CVD-RF was highest in PsA. Figure 2 shows that the number of CVD-RFs increased with advancing age and was comparable across the three IJD diseases. In addition, the presence of one to two CVD-RFs was common in the three IJD diseases and in all age strata.

**Fig. 3:**
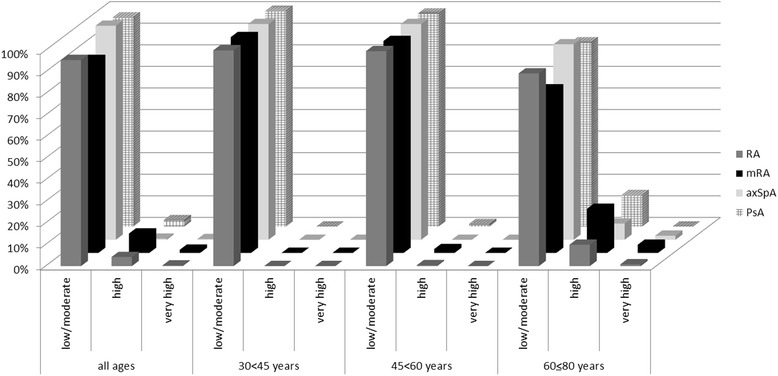
Risk of cardiovascular disease according to the Systematic Coronary Risk Evaluation (SCORE) algorithm for countries with low risk of CVD, in patients with various inflammatory joint diseases, stratified by age (30 to <45 years, 45 to <60 years and 60 to ≤80 years). *RA* rheumatoid arthritis, *mRA* modified SCORE by application of the European League Against Rheumatism 1.5 multiplication factor in patients with RA, *axSp*A axial spondyloarthritis, *PsA* psoriatic arthritis. Low to moderate risk (SCORE <5%); high risk (SCORE 5 < 10%); very high risk (SCORE ≥10%)

Among the 2530 individuals who were included, data required for CVD risk evaluation by SCORE (age, sex, smoking, sBP, TC and HDL-c) were available in 2410 (95.3%) patients. Figure 3 displays the absolute risk of future CVD stratified into risk categories across IJD entities including the 1.5 multiplication factor in RA, for different age strata. Overall, there were only small differences in the median absolute risk of CVD across IJD entities (see Additional file 1: Table S3). In the 30 to <45 and 45 to <60 age strata, almost all patients with IJD had CVD risk estimates equivalent to low to moderate risk and few had high to very high risk. However, in the oldest age stratum, there was a moderate shift towards high to very high risk (18.6% and 3.2% had high or very high risk, respectively (see Additional file 1: Table S4). The results of sensitivity analyses including only complete cases (excluding patients with missing data on CVD, diabetes mellitus, AntiHT and/or LLT), were similar (data not shown). Application of the EULAR 1.5 multiplication factor in RA patients resulted in reclassification of 16.6% of patients in the oldest age strata to a higher CVD risk category but almost none of the younger patients were reclassified (see Fig. 3 and Additional file 1: Table S4).

**Fig. 4:**
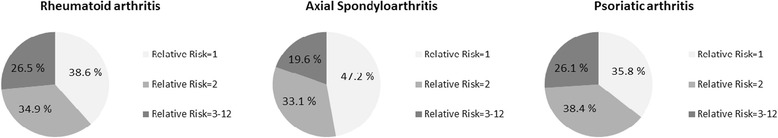
Relative risk and percentage of patients with rheumatoid arthritis, axial spondyloarthritis and psoriatic arthritis who had a relative risk corresponding to no increased risk (RR = 1), a two-fold risk (RR = 2) or a risk three times or higher (RR = 3–12) compared to individuals without cardiovascular risk factors (no smoking, systolic blood pressure ≤120 mmHg or total cholesterol ≤4 mmol/L)

In those patients in whom the absolute risk of CVD was calculated, the relative risk of future CVD was also estimated showing that 35.2% of patients with IJD had a relative risk of 2 (RR = 2), while 24.7% had a risk of future CVD three times or higher (RR = 3–12) compared to patients with optimal CVD-RF levels (see Fig. 4. and Additional file 1: Table S5).

## Discussion

Using a large cohort of patients from a nationwide project, we found a high prevalence of CVD-RFs across all the major IJD entities. CVD-RFs were also prevalent among the younger patients with IJD and there were also some interesting differences across IJD entities. Despite the high frequency of CVD-RFs, estimated CVD risk according to SCORE was low/moderate and high, and individuals at very high-risk were only identified among the oldest age stratum. Several studies have reported the frequency of certain CVD-RFs and compared patients with one particular IJD to non-IJD controls, but largely there is a lack of studies comparing the CVD risk profile across IJD entities. To date only one study has compared the prevalence of these five major CVD-RFs across all three IJD entities [13], while another study published a decennial ago compared the prevalence of three CVD-RFs (HT, lipid abnormalities and diabetes mellitus) among patients with RA, AS and PsA [14]. Moreover, these studies did not stratify patients with IJD by increasing age, which is an important determinant of CVD-RF prevalence. With the exception of one study, reports comparing estimated CVD risk by SCORE across IJD entities are also lacking. In this study, patients with documented CVD were excluded from analysis using SCORE, but it was not stated whether patients with diabetes mellitus and/or current use of LLT/AntiHT were excluded [13].

Previous studies indicate that HT is more frequent in PsA [13, 19, 20] and AS [13] than in non-IJD controls. While studies comparing HT frequency in patients with RA versus non-RA controls report inconsistent trends [13, 21, 22], two studies comparing IJD entities describe similar HT frequency in RA, AS and PsA patients [13, 14]. We observed that HT was the most prevalent CVD-RF, occurring in about half of all patients with IJD without established CVD. This is particularly interesting as patients with RA appear to be less likely to receive a diagnosis of HT than individuals without RA [23], and it appears that HT has the highest impact on CVD morbidity among the conventional CVD-RFs [9]. Furthermore, our study showed that patients with PsA were significantly more hypertensive than their counterparts with RA and axSpA within similar age strata.

Two studies report that the frequency of hyperlipidemia and hypercholesterolemia diagnoses, respectively, was comparable across IJD entities [13, 14]. Likewise, we did not observe any significant differences across IJD entities in the prevalence of elevated TC levels. However, a significant age effect was present. The high frequency of elevated TC levels is of particular interest as previous studies indicate that patients with RA are rarely screened for lipid abnormalities [24], and that patients with RA [6, 25], and with axSpA or PsA [6], are undertreated in terms of lipid-associated CVD risk.

In addition to being an important CVD-RF, obesity is a risk factor for developing IJD, especially PsA [26]. In line with findings in Spanish patients with IJD [13], we also observed that obesity was more frequent in PsA compared to the other IJD entities within the two youngest age strata. These findings are in line with obesity being a particularly strong risk factor for PsA. However, the prevalence of obesity among patients with IJD (17.4%) did not differ from estimations in the general population (17–20%) by the Norwegian Institute of Public Health [27]. The clinical relevance of the attention focused on obesity is evident, since managing obesity may not only improve CVD risk but also rheumatic disease activity and response to antirheumatic treatment [28].

Smoking is an established risk factor for developing RA [29], PsA [30] and AS [31] and possibly induces autoimmunity by triggering citrullination of peptides [32]. Although smoking was not as frequent in this study as previously reported in Spanish patients with IJD and Danish patients with RA [13, 33], we identified a prevalence of 20.1% of daily smokers among our patients with IJD, which is higher than the 13% of reported smokers among the general Norwegian population [34]. Fortunately, daily smoking appears less frequent in younger patients with IJD.

The prevalence of diabetes mellitus has been reported to be significantly higher in patients with PsA compared to those with RA [35]. However, we found no statistically significant differences in the frequency of diabetes mellitus across IJD entities except a borderline significantly higher frequency of diabetes mellitus among younger patients with RA. Furthermore, the overall prevalence of diabetes mellitus among our patients was much lower than in the patients studied by Labitigan et al. [35]. Although the prevalence of diabetes mellitus among Norwegian patients with IJD (4.8–6.0%) was close to that estimated in the Norwegian general population (4.3%) [27], there are indications that diabetes mellitus is under-reported in IJD (e.g. in RA) [36].

Moreover, we showed that modifiable CVD-RFs were common across all IJD entities and even among patients as young as 30 to <45 years. Although CVD-RF prevalence and CVD-RF quantity were highly age-dependent, there were also some significant differences between IJD entities. Patients with PsA appeared to have higher numbers of CVD-RFs due to frequent HT and/or obesity. These findings are in line with obesity being a particularly strong risk factor for development of PsA [26], and furthermore, the association between obesity and HT [37].

Despite a high frequency of CVD-RFs in patients with IJD, we found that overall the patients with IJD included in the analysis had a low estimated CVD risk according to SCORE. A high or very high CVD risk according to SCORE was almost exclusively found in the oldest patients with IJD. Interestingly, according to relative risk estimations, there was a high frequency of patients with RA, axSpA or PsA who had twofold or even higher risk compared to individuals with no CVD-RFs. This illustrates that patients with IJD have a high relative risk of CVD despite a low absolute risk.

Previous studies indicate that the SCORE algorithm generally underestimates CVD risk in patients with RA [11], and a great number of patients with RA with moderate CVD risk according to SCORE have carotid atherosclerosis on ultrasound [38]. In the latest ESC guidelines, atherosclerotic plaque(s) identified by carotid ultrasound are considered an equivalent risk to a diagnosis of CVD [4]. Application of the EULAR 1.5 multiplication factor in patients with RA did not result in a significant clinical reclassification into a higher CVD risk category except for a few of the oldest patients (see Fig. 3 and Additional file 1: Table S4).

To our knowledge, only one previous study has reported on the prevalence of these five CVD-RFs across IJD entities. However, this is the first project also reporting on the distribution of CVD-RFs in relation to age strata. Furthermore, in contrast to a previous publication [13], we explicitly excluded patients with diabetes mellitus and/or use of LLT and AntiHT, when estimating CVD risk according to the SCORE algorithm. One of the strengths of our study is that relatively large numbers of patients were included in this nationwide project, which supports the generalizability of our findings. Our investigation of CVD-RF prevalence has high clinical relevance as the CVD-RFs included in the analysis account for half of all CVD morbidity and mortality [8, 9]. Furthermore, we have clearly defined the CVD-RFs investigated. Lasty, since age is strongly associated with CVD risk and CVD-RF prevalence, we have estimated their distribution in different age strata.

Several study limitations should be considered when interpreting these results. First, selection bias is indicated by the high rate of use of bDMARDs (especially in axSpA) and the inclusion rate in NOCAR of 41% among all eligible patients [6]. Patients included in NOCAR appear to have had lower disease activity at the time of inclusion and, overall, older age compared to eligible but non-included patients [6]. Since high disease activity is associated with increased risk of CVD [39], our results may thus underestimate the actual CVD risk in patients with IJD with high disease activity. Second, missing and incomplete data are another limitation. Unfortunately, due to poor reporting, nonsteroidal anti-inflammatory drug (NSAID) use was excluded from the analysis. There was also incomplete reporting on LLT use (see Additional file 1: Table S6). Since patients with incomplete reporting on atherosclerotic CVD comorbidities (MI, PCI, CABG, stroke and/or TIA), were classified as patients without CVD, the prevalence of established CVD may have been underestimated. Although self-reported risk factors may be prone to systematic report errors, self-reported tobacco use has previously been shown to be a valid marker for tobacco exposure [40], and self-reported information on statins and antihypertensive medications has been shown to have high agreement with pharmacy records [41]. Furthermore, any recorded data are subject to errors of coding or misclassification. However, we do not believe that any bias would disproportionately affect one IJD entity more than the others. Due to the absence of a control group, we could not compare the CVD risk profile in individuals with and without IJD. Last, as we report findings from a nationwide cohort from a country with low risk of CVD consisting of a predominantly Caucasian population, the external validity outside Norway/Nordic countries is somewhat uncertain.

## Conclusions

In conclusion we have shown for the first time that the frequency of conventional CVD-RFs is just as high in patients with axSpA and PsA as in RA. Patients with PsA even appear to have more frequent hypertension and obesity, which is not assessed by the SCORE CVD risk algorithm. Despite most patients with IJD having a low/moderate absolute risk of CVD, the relative risk was frequently increased. Our findings underscore the need for CVD risk assessment in all patients with IJD.

## Additional file

Additional file 1:
**Table S1** Sensitivity analyses of prevalence of cardiovascular disease risk factors across age strata in patients with rheumatoid arthritis, axial spondylitis and psoriatic arthritis. **Table S2** Quantity of conventional cardiovascular risk factors. **Table S3** Ten year risk of fatal cardiovascular disease events. **Table S4** Cardiovascular risk categories. **Table S5** Relative risk in patients with inflammatory joint diseases. **Table S6** Data availability for cardiovascular risk factors. **Table S7** Patient characteristics and cardiovascular risk factors according to rheumatic disease activity. **Table S8** Characteristics of rheumatoid arthritis patients according to rheumatoid factor and anti-citrullinated protein antibody positivity. **Table S9** Patient characteristics and cardiovascular risk factors according to current use of biologic agents. (DOCX 58 kb)

## Abbreviations

ACPA: Anti-citrullinated protein antibody
ANOVA: Analysis of variance
AntiHT: Antihypertensive therapy
axSpA: Axial spondyloarthritis
BMI: Body mass index
BP: Blood pressure
CABG: Coronary artery bypass graft
CVD: Cardiovascular disease
CVD-RF: Cardiovascular disease risk factor
dBP: Diastolic blood pressure
ESC: European Society of Cardiology
EULAR: European League Against Rheumatism
HDL-c: High density lipoprotein-cholesterol
HT: Hypertension
IJD: Inflammatory joint diseases
LDL-c: Low density lipoprotein-cholesterol
LLT: Lipid-lowering therapy
MI: Myocardial infarction
NHANES: National Health and Nutrition Examination Survey
NOCAR: Norwegian Collaboration on Atherosclerotic disease in patients with Rheumatic joint diseases
PAD: Peripheral artery disease
PCI: Percutaneous coronary intervention
PsA: Psoriatic arthritis
RA: Rheumatoid arthritis
RF: Rheumatoid factor
RR: Relative risk
sBP: Systolic blood pressure
SCORE: Systematic coronary risk evaluation
sDMARDs: Synthetic disease-modifying antirheumatic drugs
TC: Total cholesterol
TG: Triglycerides
TIA: Transient ischemic attack
