# Supplementary material for: Cardiovascular disease risk profiles in inflammatory joint disease entities
Source: Arthritis Res Ther. 2017 Jul 3;19:153. doi: 10.1186/s13075-017-1358-1 (PMC5496163; doi:10.1186/s13075-017-1358-1)
Supplement: Additional file 1: — Table S1 Sensitivity analyses of prevalence of cardiovascular disease risk factors across age strata in patients with rheumatoid arthritis, axial spondylitis and psoriatic arthritis. Table S2 Quantity of conventional cardiovascular risk factors. Table S3 Ten year risk of fatal cardiovascular disease events. Table S4 Cardiovascular risk categories. Table S5 Relative risk in patients with inflammatory joint diseases. Table S6 Data availability for cardiovascular risk factors. Table S7 Patient characteristics and cardiovascular risk factors according to rheumatic disease activity. Table S8 Characteristics of rheumatoid arthritis patients according to rheumatoid factor and anti-citrullinated protein antibody positivity. Table S9 Patient characteristics and cardiovascular risk factors according to current use of biologic agents. (DOCX 58 kb) [file 13075_2017_1358_MOESM1_ESM.docx]

**Additional files**

**Table S1.** Sensitivity analyses of prevalence of cardiovascular disease risk factors across age strata in patients with rheumatoid arthritis, axial spondylitis and psoriatic arthritis

|  |  | **Inflammatory**  **joint disease (n=3517)** | **Rheumatoid arthritis**  **(n=1961)** | **Axial Spondyloarthritis**  **(n=835)** | **Psoriatic**  **arthritis**  **(n=721)** | **p** |
| --- | --- | --- | --- | --- | --- | --- |
| Hypertension | Valid observations | 3514 (99.9) | 1959 (99.9) | 834 (99.9) | 721(100) | - |
| Any ages, n (%) | Percent  Valid percent | 1751 (49.8)  1751 (49.8) | 1003 (51.1)  1003 (51.2) | 348 (41.7)  348 (41.7) | 400 (55.5)  400 (55.5) | **<0.001** |
| Age 30<45 years, n (%) | Percent  Valid percent | 217 (28.0)  217 (28.0) | 57 (23.3)  57 (23.3) | 89 (26.3)  89 (26.3) | 71 (37.2)  71 (48.6) | **<0.001** |
| Age 45<60 years, n (%) | Percent  Valid percent | 699 (47.1)  699 (47.1) | 305 (41.8)  305 (41.9) | 182 (47.3)  182 (47.4) | 212 (57.1)  212 (57.1) | **<0.001** |
| Age 60-80 years, n (%) | Percent  Valid percent | 835 (66.4)  835 (66.4) | 641 (64.9)  641 (65.0) | 77 (68.8)  77 (68.8) | 117 (73.6)  117 (73.6) | 0.090 |
| Elevated total cholesterol | Valid observations | 3486 (99.1) | 1945 (99.2) | 828 (99.2) | 713 (98.9) | - |
| Any ages, n (%) | Percent  Valid percent | 1152 (32.8)  1152 (33.0) | 711 (36.3)  711 (36.6) | 221 (26.5)  221 (26.7) | 220 (30.5)  220 (30.9) | **<0.001** |
| Age 30<45 years, n (%) | Percent  Valid percent | 110 (14.2)  110 (14.4) | 29 (11.8)  29 (11.8) | 55 (16.3)  55 (16.5) | 26 (13.6)  26 (14.0) | 0.293 |
| Age 45<60 years, n (%) | Percent  Valid percent | 490 (33.0)  490 (33.2) | 243 (33.3)  243 (33.6) | 118 (30.6)  118 (30.7) | 129 (34.8)  129 (35.0) | 0.442 |
| Age 60-80 years, n (%) | Percent  Valid percent | 552 (43.9)  552 (44.3) | 439 (44.5)  439 (44.9) | 48 (42.9)  48 (43.6) | 65 (40.9)  65 (41.1) | 0.658 |
| Obesity | Valid observations | 3164 (90.0) | 1816 (92.6) | 739 (88.5) | 609 (84.5) | - |
| Any ages, n (%) | Percent  Valid percent | 613 (17.4)  613 (19.4) | 315 (16.1)  315 (17.3) | 133 (15.9)  133 (18.0) | 165 (22.9)  165 (27.1) | **<0.001** |
| Age 30<45 years, n (%) | Percent  Valid percent | 150 (19.4)  150 (21.8) | 49 (20.0)  49 (21.6) | 52 (15.4)  52 (17.4) | 49 (25.7)  49 (30.4) | **<0.001** |
| Age 45<60 years, n (%) | Percent  Valid percent | 269 (18.1)  269 (20.4) | 120 (16.5)  120 (17.9) | 63 (16.4)  63 (18.6) | 86 (23.2)  86 (28.0) | **<0.001** |
| Age 60-80 years, n (%) | Percent  Valid percent | 194 (15.4)  194 (16.7) | 146 (14.8)  146 (15.9) | 18 (16.1)  18 (17.8) | 30 (18.9)  30 (21.3) | 0.271 |
| Current smoking | Valid observations | 3472 (98.7) | 1938 (98.8) | 820 (98.2) | 714 (99.0) | - |
| Any ages, n (%) | Percent  Valid percent | 707 (20.1)  707 (20.4) | 419 (21.4)  419 (21.6) | 162 (19.4)  162 (19.8) | 126 (17.5)  126 (17.6) | 0.070 |
| Age 30<45 years, n (%) | Percent  Valid percent | 125 (16.1)  125 (16.3) | 39 (15.9)  39 (16.0) | 59 (17.5)  59 (17.6) | 27 (14.1)  27 (14.2) | 0.070 |
| Age 45<60 years, n (%) | Percent  Valid percent | 317 (21.3)  317 (21.6) | 161 (22.1)  161 (22.3) | 77 (20.0)  77 (20.4) | 79 (21.3)  79 (21.4) | 0.758 |
| Age 60-80 years, n (%) | Percent  Valid percent | 265 (21.1)  265 (21.5) | 219 (22.2)  219 (22.5) | 26 (23.2)  26 (24.3) | 20 (12.6)  20 (12.9) | **0.019** |
| Diabetes | Valid observations | 2815 (80.0) | 1626 (82.9) | 635 (76.0) | 554 (76.8) | - |
| Any ages, n (%) | Percent  Valid percent | 169 (4.8)  169 (6.0) | 98 (5.0)  98 (6.0) | 28 (3.4)  28 (4.4) | 43 (6.0)  43 (7.8) | 0.052 |
| Age 30<45 years, n (%) | Percent  Valid percent | 28 (3.6)  28 (4.3) | 13 (5.3)  13 (6.2) | 7 (2.1)  7 (2.5) | 8 (4.2)  8 (5.0) | 0.052 |
| Age 45<60 years, n (%) | Percent  Valid percent | 69 (4.6)  69 (6.1) | 33 (4.5)  33 (5.6) | 16 (4.2)  16 (5.7) | 20 (5.4)  20 (7.6) | 0.493 |
| Age 60-80 years, n (%) | Percent  Valid percent | 72 (5.7)  72 (7.0) | 52 (5.3)  52 (6.3) | 5 (4.5)  5 (6.4) | 15 (9.4)  15 (11.5)) | 0.098 |

*Percent: prevalence of cardiovascular risk factors among all included patients; Valid percent: prevalence of cardiovascular risk factors in patients with complete recording of the particular risk factor*

**Table S2.** Quantity of conventional cardiovascular risk factors

| **Age** | **Number of**  **CVD-RFs** | **Inflammatory**  **joint disease**  **(n=3517)** | **Rheumatoid**  **arthritis**  **(n=1961)** | **Axial**  **spondyloarthritis (n=835)** | **Psoriatic**  **arthritis**  **(n=721)** | **p** |
| --- | --- | --- | --- | --- | --- | --- |
| Any | Minimum 1, n (%) | 2587 (73.6) | 1492 (76.1) | 538 (64.4) | 557 (77.3) | **<0.001** |
|  | Minimum 2, n (%) | 1324 (37.4) | 776 (39.6) | 251 (30.1) | 287 (39.8) | **<0.001** |
|  | Minimum 3, n (%) | 416 (11.8) | 242 (12.3) | 84 (10.1) | 90 (12.5) | 0.191 |
|  | Minimum 4, n (%) | 56 (1.6) | 30 (1.5) | 11 (1.3) | 15 (2.1) | 0.457 |
|  | Five of five, n (%) | 5 (0.1) | 2 (0.1) | 1 (0.1) | 2 (0.3) | 0.412 |
| 30<45 years | Minimum 1, n (%) | 412 (53.2) | 126 (51.4) | 167 (49.4) | 119 (62.3) | **0.013** |
|  | Minimum 2, n (%) | 158 (20.4) | 45 (18.4) | 68 (20.1) | 45 (23.6) | 0.403 |
|  | Minimum 3, n (%) | 45 (5.8) | 13 (5.3) | 20 (5.9) | 12 (6.3) | 0.923 |
|  | Minimum 4, n (%) | 8 (1.0) | 3 (1.2) | 2 (0.6) | 3 (1.6) | 0.488 |
|  | Five of five, n (%) | 1 (0.1) | 0 (0) | 1 (0.3) | 0 (0) | 1.00 |
| 45<60 years | Minimum 1, n (%) | 1091 (73.5) | 518 (71.1) | 274 (71.2) | 299 (80.6) | **0.002** |
|  | Minimum 2, n (%) | 555 (37.4) | 257 (35.3) | 130 (33.8) | 168 (45.3) | **0.001** |
|  | Minimum 3, n (%) | 170 (11.4) | 77 (10.6) | 42 (10.9) | 51(13.7) | 0.270 |
|  | Minimum 4, n (%) | 20 (1.3) | 7 (1.0) | 7 (1.8) | 6 (1.6) | 0.391 |
|  | Five of five, n (%) | 2 (0.1) | 1 (0.1) | 0 (0) | 1 (0.3) | 0.745 |
| 60-80 years | Minimum 1, n (%) | 1084 (86.2) | 848 (85.9) | 97 (86.6) | 139 (87.4) | 0.855 |
|  | Minimum 2, n (%) | 601 (47.8) | 474 (48.0) | 53 (47.3) | 74 (46.5) | 0.933 |
|  | Minimum 3, n (%) | 201 (16.0) | 152 (15.4) | 22(19.6) | 27(17.0) | 0.481 |
|  | Minimum 4, n (%) | 28 (2.2) | 20 (2.0) | 2 (1.8) | 6 (3.8) | 0.364 |
|  | Five of five, n (%) | 2 (0.2) | 1 (0.1) | 0 (0) | 1 (0.6) | 0.385 |

*Numbers of cardiovascular risk factors across inflammatory joint disease entities and age strata. CVD-RFs: Cardiovascular disease risk factors.*

**Table S3.** Ten year risk of fatal cardiovascular disease events

| **SCORE** | **IJD (mRA)**  **(n=2530)** | **IJD**  **(n=2530)** | **RA**  **(n=1354)** | **mRA**  **(n=1354)** | **axSpA**  **(n=646)** | **PsA**  **(n=530)** | **p**  **(RA)** | **p**  **(mRA)** |
| --- | --- | --- | --- | --- | --- | --- | --- | --- |
| All ages |  |  |  |  |  |  | - | - |
| Numbers (valid observations) | 2410/2530 (95.3) | 2410/2530 (95.3) | 1293/1354 (95.5) | 1293/1354 (95.5) | 613/646  (94.9) | 504/530  (95.1) | - | - |
| SCORE, median (IQR) | 0.75  (0.30, 1.95) | 0.60  (0.20, 1.50) | 0.80  (0.20, 1.8) | 1.20  (0.30, 2.70) | 0.40  (0.20, 1.00) | 0.60  (0.20, 1.30) | **<0.001** | **<0.001** |
| Age 30<45 years |  |  |  |  |  |  | - | - |
| Numbers (valid observations) | 677/702  (96.4) | 677/702  (96.4) | 215/220  (97.7) | 215/220  (97.7) | 297/309  (96.1) | 165/173  (95.4) | - | - |
| SCORE, median (IQR) | 0.20  (0.0, 0.30) | 0.20  (0.00, 0.30) | 0.10  (0.00, 0.10) | 0.15  (0.00, 0.15) | 0.20  (0.10, 0.40) | 0.20  (0.10, 0.40) | **<0.001** | **<0.001** |
| Age 45<60 years |  |  |  |  |  |  | - | - |
| Numbers (valid observations) | 1073/1120 (95.8) | 1073/1120 (95.8) | 553/572  (96.7) | 553/572  (96.7) | 263/280  (93.9) | 257/268  (95.9) | - | - |
| SCORE, median (IQR) | 0.75  (0.30, 1.50) | 0.60  (0.30, 1.20) | 0.50  (0.20, 1.10) | 0.75  (0.30, 1.65) | 0.80  (0.40, 1.40) | 0.80  (0.30, 1.40) | **<0.001** | 0.866 |
| Age 60<80 years |  |  |  |  |  |  | - | - |
| Numbers (valid observations) | 660/708  (93.2) | 660/708  (93.2) | 525/562  (93.4) | 525/562  (93.4) | 53/57  (93.0) | 82/89  (92.1) | - | - |
| SCORE, median (IQR) | 2.70  (1.60, 4.65) | 2.00  (1.10, 3.40) | 1.90  (1.10, 3.30) | 2.85  (1.65, 4.95) | 2.30  (1.30, 4.25) | 2.30  (1.20, 3.93) | 0.121 | **0.006** |

*Ten year risk of fatal cardiovascular disease according to Systematic Coronary Risk Evaluation across age strata and inflammatory joint disease entities. SCORE: Systematic Coronary Risk Evaluation; IJD: inflammatory joint diseases, RA: rheumatoid arthritis; mRA: modified SCORE using the EULAR 1.5 multiplication factor on estimated risk in RA; axSpA: axial spondyloarthritis; PsA: psoriatic arthritis, IQR: Interquartile range*

**Table S4.** Cardiovascular risk categories

|  | **IJD (mRA)**  **(n=2530)** | **IJD**  **(n=2530)** | **RA**  **(n=1354)** | **mRA**  **(n=1354)** | **axSpA**  **(n=646)** | **PsA**  **(n=530)** |
| --- | --- | --- | --- | --- | --- | --- |
| All ages |  |  |  |  |  |  |
| Numbers (valid observations) | 2410/2530  (95.3) | 2410/2530  (95.3) | 1293/1354  (95.5) | 1293/1354  (95.5) | 613/646  (94.9) | 504/530  (95.1) |
| Low-to-moderate risk (<5%), n (%) | 2253 (93.5) | 2332 (96.8) | 1235 (95.5) | 1156 (89.4) | 608 (99.2) | 489 (97.0) |
| High risk (5<10%) n (%) | 136 (5.6) | 73 (3.0) | 54 (4.2) | 117 (9.0) | 4 (0.7) | 15 (3.0) |
| Very high risk (>10%), n (%) | 21 (0.9) | 5 (0.2) | 4 (0.3) | 20 (1.5) | 1 (0.2) | 0 (0) |
| Reclassified, n (%) | - | - | - | 95 (7.3) | - | - |
| Age 30<45 |  |  |  |  |  |  |
| Numbers (valid observations) | 677/702  (96.4) | 677/702  (96.4) | 215/220  (97.7) | 215/220  (97.7) | 297/309  (96.1) | 165/173  (95.4) |
| Low-to-moderate risk (<5%), n (%) | 677 (100) | 677 (100) | 215 (100) | 215 (100) | 297 (100) | 165 (100) |
| High risk (5<10%), n (%) | 0 (0) | 0 (0) | 0 (0) | 0 (0) | 0 (0) | 0 (0) |
| Very high risk (>10%), n (%) | 0 (0) | 0 (0) | 0 (0) | 0 (0) | 0 (0) | 0 (0) |
| Reclassified, n (%) | - | - | - | 0 (0) | - | - |
| Age 45<60 |  |  |  |  |  |  |
| Numbers (valid observations) | 1073/1120  (95.8) | 1073/1120  (95.8) | 553/572  (96.7) | 553/572  (96.7) | 263/280  (93.9) | 257/268  (95.9) |
| Low-to-moderate risk (<5%), n (%) | 1060 (98.8) | 1068 (99.5) | 551 (99.6) | 543 (98.2) | 263 (100) | 254 (98.8) |
| High risk (5<10%), n (%) | 13 (1.2) | 5 (0.5) | 2 (0.4) | 10 (1.8) | 0 (0) | 3 (1.2) |
| Very high risk (>10%), n (%) | 0 (0) | 0 (0) | 0 (0) | 0 (0) | 0 (0) | 0 (0) |
| Reclassified, n (%) | - | - | - | 8 (1.4) | - | - |
| Age 60<80 |  |  |  |  |  |  |
| Numbers (valid observations) | 660/708  (93.2) | 660/708  (93.2) | 525/562  (93.4) | 525/562  (93.4) | 53/57  (93.0) | 82/89  (92.1) |
| Low-to-moderate risk (<5%), n (%) | 516 (78.2) | 587 (88.9) | 469 (89.3) | 398 (75.8) | 48 (90.6) | 70 (85.4) |
| High risk (5<10%), n (%) | 123 (18.6) | 68 (10.3) | 52 (9.9) | 107 (20.4) | 4 (7.5) | 12 (14.6) |
| Very high risk (>10%), n (%) | 21 (3.2) | 5 (0.8) | 4 (0.8) | 20 (3.8) | 1 (1.9) | 0 (0) |
| Reclassified, n (%) | - | - | - | 87 (16.6) | - | - |

*Ten year risk of fatal cardiovascular disease according to the Systematic Coronary Risk Evaluation. IJD: inflammatory joint diseases, RA: rheumatoid arthritis; mRA: modified SCORE using the EULAR 1.5 multiplication factor on estimated risk in RA; axSpA: axial spondyloarthritis; PsA: psoriatic arthritis.*

**Table S5**. Relative Risk in patients with inflammatory joint diseases

| ***Relative risk 1-12*** | ***IJD***  ***(n=2530)*** | ***RA***  ***(n=1354)*** | ***axSpA***  ***(n=646)*** | ***PsA***  ***(n=530)*** | ***p*** |
| --- | --- | --- | --- | --- | --- |
| Valid observations, n (%) | 2422 (95.7) | 1301 (96.1) | 616 (95.4) | 505 (95.3) | - |
| Relative risk, median (IQR) | 1 (1, 2) | 2 (1, 3) | 2 (1, 2) | 2 (1, 3) | **<0.001** |
| Relative risk= 1 | 972 (40.1) | 502 (38.6) | 291 (47.2) | 179 (35.8) | - |
| Relative risk= 2 | 852 (35.2) | 454 (34.9) | 204 (33.1) | 194 (38.4) | - |
| Relative risk= 3-12 | 598 (24.7) | 345 (26.5) | 121 (19.6) | 132 (26.1) | - |
| *Relative risk= 3* | *369 (15.2)* | *207 (15.9)* | *76 (12.3)* | *86 (17.0)* | *-* |
| *Relative risk= 4* | *128 (5.3)* | *73 (5.6)* | *26 (4.2)* | *29 (5.7)* | *-* |
| *Relative risk= 5* | *49 (2.0)* | *34 (2.6)* | *4 (0.6)* | *11 (2.2)* | *-* |
| *Relative risk= 6* | *26 (1.1)* | *14 (1.1)* | *10 (1.6)* | *2 (0.4)* | *-* |
| *Relative risk= 7* | *13 (0.5)* | *9 (0.7)* | *2 (0.3)* | *2 (0.4)* | *-* |
| *Relative risk= 8* | *11 (0.5)* | *6 (0.5)* | *3 (0.5)* | *2 (0.4)* | *-* |
| *Relative risk= 10* | *1 (0.0)* | *1 (0.1)* | *0 (0)* | *0 (0)* | *-* |
| *Relative risk= 12* | *1 (0.0)* | *1 (0.1)* | *0 (0)* | *0 (0)* | *-* |

*Relative risk: ratio comparing absolute risk in individuals compared to risk given optimal levels of cardiovascular risk factors in patients with inflammatory joint diseases (IJD), RA: rheumatoid arthritis, axSpA: axial spondyloarthritis, PsA: psoriatic arthritis, IQR: inter-quartile range.***Table S6*.*** *Data-availability of cardiovascular risk factors*

| **Observations of cardiovascular risk factors** | **IJD**  **(n=3517)** | **RA**  **(n=1961)** | **axSpA (n=835)** | **PsA**  **(n=721)** |
| --- | --- | --- | --- | --- |
| **Hypertension** |  |  |  |  |
| *Self-reported HTN, AntiHT status, sBP and/or dBP level* | 3514 (99.9) | 1959 (99.9) | 834 (99.9) | 721 (100) |
| *Self-reported HTN, AntiHT status, sBP and dBP level* | 2201 (62.6) | 1262 (64.4) | 519 (62.2) | 420 (58.3) |
| *Status of self-reported HTN* | 2790 (79.3) | 1608 (82.0) | 633 (75.8) | 549 (76.1) |
| *AntiHT status* | 2717 (77.3) | 1458 (74.3) | 698 (83.6) | 561 (77.8) |
| *sBP level* | 3483 (99.0) | 1942 (99.0) | 825 (98.8) | 716 (99.3) |
| *dBP level* | 3482 (99.0) | 1943 (99.1) | 824 (98.7) | 715 (99.2) |
| **Elevated total cholesterol** |  |  |  |  |
| *Status of lipid-lowering therapy and/or total cholesterol* | 3486 (99.1) | 1945 (99.2) | 828 (99.2) | 713 (98.9) |
| *Status of lipid-lowering therapy and total cholesterol* | 2615 (74.4) | 1401 (71.4) | 675 (80.8) | 539 (74.8) |
| *Status of lipid-lowering therapy* | 2646 (75.2) | 1417 (72.3) | 682 (81.7) | 547 (75.9) |
| *Total cholesterol* | 3455 (98.2) | 1929 (98.4) | 821 (98.3) | 705 (97.8) |
| **Diabetes mellitus** |  |  |  |  |
| *Status of diabetes mellitus* | 2815 (80.0) | 1626 (82.9) | 635 (76.0) | 554 (76.8) |
| **Obesity** |  |  |  |  |
| *Body Mass Index* | 3164 (90.0) | 1816 (92.6) | 739 (88.5) | 609 (84.5) |
| **Current smoking** |  |  |  |  |
| *Status of current smoking* | 3472 (98.7) | 1938 (98.8) | 820 (98.2) | 714 (99.0) |

*Numbers and percent of available data on presence of hypertension, elevated total cholesterol, diabetes mellitus, obesity and current smoking. IJD: inflammatory joint disease; RA: rheumatoid arthritis; axSpA: axial spondyloarthritis; PsA: psoriatic arthritis; HTN: hypertension, AntiHT: antihypertensive treatment; sBP and dBP: systolic and diastolic blood pressure.*

**Table S7**. Patient characteristics and cardiovascular risk factors according to rheumatic disease activity

| **Variables** | **Rheumatoid arthritis**  **DAS28 disease activity** | |  | | **Axial spondyloarthritis**  **ASDAS disease activity** | |  | |
| --- | --- | --- | --- | --- | --- | --- | --- | --- |
|  | **Remission/Low**  **(n=1185)** | **High/Very high**  **(n=468)** | | **p** | **Inactive/ Moderate**  **(n=401)** | **High/ Very high**  **(n=224)** | | **p** |
| Females, n (%) | 804 (67.8) | 362 (77.4) | | **<0.001** | 131 (32.7) | 83 (37.1) | | 0.268 |
| Age in years, mean +SD | 58.7+11.1 | 59.9+11.1 | | **0.043** | 47.4 +9.7 | 49.2+9.6 | | **0.021** |
| Age 30<45 years, n (%)  Age 45<60 years, n (%)  Age 60<80 years, n (%) | 153 (12.9)  446 (37.6)  586 (49.5) | 50 (10.7)  165 (35.3)  253 (54.1) | | -  -  - | 184 (45.9)  170 (42.4)  47 (11.7) | 79 (35.3)  112 (50.0)  33 (14.7) | | -  -  - |
| Working/student, n (%) | 539 (47.1) | 149 (32.7) | | **<0.001** | 301 (77.4) | 118 (54.4) | | **<0.001** |
| Education in years, mean+SD | 12.4+3.3 | 12.3+3.3 | | 0.323 | 14.2+3.1 | 13.1+3.0 | | **<0.001** |
| **Cardiovascular risk factor levels** |  |  | |  |  |  | |  |
| Total cholesterol (mmol/l), mean+SD | 5.41+1.06 | 5.39+1.14 | | 0.783 | 5.25+1.03 | 5.27+1.08 | | 0.818 |
| Triglycerides (mmol/l), median (IQR) | 1.25 (0.91, 1.74) | 1.24 (0.96, 1.76) | | 0.565 | 1.28 (0.89, 1.88) | 1.40 (1.00, 2.06) | | **0.038** |
| Low density lipoprotein (mmol/l), mean+SD | 3.30+0.95 | 3.23+1.03 | | 0.211 | 3.17+0.96 | 3.34+0.92 | | **0.039** |
| High density lipoprotein (mmol/l), mean+SD | 1.64+0.52 | 1.64+0.52 | | 0.931 | 1.49+0.48 | 1.40+0.41 | | **0.024** |
| Body mass index (kg/m^2^), mean+SD | 26.05+4.40 | 25.99+4.69 | | 0.811 | 26.2+4.2 | 26.6+4.4 | | 0.308 |
| Systolic blood pressure (mmHg), mean+SD | 131.6+17.3 | 133.0+17.5 | | 0.154 | 128.5+15.8 | 130.9+18.2 | | 0.094 |
| Diastolic blood pressure (mmHg), mean+SD | 79.7+9.2 | 80.5+9.2 | | 0.090 | 80.2+9.6 | 81.1+10.5 | | 0.293 |
| **Prevalence of cardiovascular risk factors** |  |  | |  |  |  | |  |
| Elevated total cholesterol, n (%) | 422 (35.6) | 182 (38.9) | | 0.408 | 102 (25.4) | 57 (25.4) | | 0.767 |
| Hypertension, n (%) | 581 (49.0) | 256 (54.7) | | **0.038** | 149 (37.2) | 108 (48.2) | | **0.007** |
| Obesity, n (%) | 178 (16.1) | 86 (19.2) | | 0.139 | 59 (16.3) | 42 (22.2) | | 0.088 |
| Current smoking, n (%) | 260 (22.1) | 97 (20.9) | | 0.575 | 67 (16.9) | 53 (24.7) | | **0.026** |
| Diabetes, n (%) | 66 (6.3) | 22 (5.6) | | 0.656 | 13 (3.7) | 11 (6.6) | | 0.135 |
| **Rheumatic disease related variables** |  |  | |  |  |  | |  |
| Rheumatoid Factor+, n (%)  Anti-citrullinated protein antibody+, n (%) | 490 (68.2)  635 (78.6) | 201 (63.8)  273 (76.0) | | 0.172  0.334 | -  - | -  - | | -  - |
| Human leukocyte antigen B27+, n (%) | - | - | | - | 246 (90.8) | 98 (79.0) | | <0.001 |
| Disease duration (years), median (IQR) | 8.0 (4.0, 14.3) | 8.6 (3.3, 16.3) | | 0.530 | 10.3 (4.1, 20.5) | 10.0 (3.4, 22.1) | | 0.940 |
| ESR (mm/h), median (IQR) | 8 (4, 15) | 19 (12, 30) | | **<0.001** | 6 (3, 11) | 9 (4, 21) | | **<0.001** |
| CRP (mg/l), median (IQR) | 2 (1, 5) | 5 (2, 11) | | **<0.001** | 1 (1, 3) | 5 (3, 10) | | **<0.001** |
| **Antirheumatic medication, current use** |  |  | |  |  |  | |  |
| Glucocorticoids, n (%) | 337 (28.4) | 151 (32.3) | | 0.124 | 8 (2.0) | 3 (1.3) | | 0.550 |
| Methotrexate, n (%) | 725 (61.2) | 224 (47.9) | | **<0.001** | 28 (7.0) | 14 (6.3) | | 0.726 |
| Other sDMARDs, n (%) | 908 (76.6) | 334 (71.4) | | **0.026** | 43 (10.7) | 25 (11.2) | | 0.866 |
| bDMARDs, n (%) | 515 (43.5) | 189 (40.4) | | 0.255 | 282 (70.3) | 128 (57.1) | | **<0.001** |

*Patient characteristics and cardiovascular risk factors according to status of rheumatic disease activity. DAS28: Disease activity score using 28 joint count; ASDAS: Ankylosing spondylitis disease activity score; SD: standard deviation; ESR: erythrocyte sedimentation rate; CRP: C-reactive protein; sDMARDs and bDMARDs: synthetic and biologic disease-modifying antirheumatic drugs.*

**Table S8.** Characteristics of rheumatoid arthritis patients according to rheumatoid factor and anti-citrullinated protein antibody positivity

| **Variables** | **ACPA+**  **(n=1066)** | **ACPA-**  **(n=313)** | **p** | **RF+**  **(n=787)** | **RF-**  **(n=397)** | **p** |
| --- | --- | --- | --- | --- | --- | --- |
| Females, n (%) | 757 (71.0) | 220 (70.3) | 0.804 | 554 (70.4) | 278 (70.0) | 0.896 |
| Age in years, mean+SD | 58.2+11.2 | 58.8+11.4 | 0.403 | 58.8+11.4 | 58.5+11.4 | 0.659 |
| Age 30<45 years, n (%)  Age 45<60 years, n (%)  Age 60<80 years, n (%) | 148 (13.9)  412 (38.6)  506 (47.5) | 38 (12.1)  114 (36.4)  161 (51.4) | -  -  - | 106 (13.5)  280 (35.6)  401 (51.0) | 53 (13.4)  148 (37.3)  196 (49.4) | -  -  - |
| Working/student, n (%) | 464 (46.0) | 126 (41.6) | 0.172 | 326 (43.5) | 171 (45.0) | 0.624 |
| Education in years, mean+SD | 12.4+3.4 | 12.6+3.3 | 0.452 | 12.3+3.4 | 12.7+3.3 | 0.089 |
| **CVD risk factor levels** |  |  |  |  |  |  |
| Total cholesterol (mmol/l), mean+SD | 5.36+1.10 | 5.55+1.13 | **0.008** | 5.39+1.12 | 5.41+1.11 | 0.780 |
| Triglycerides (mmol/l), median (IQR) | 1.20 (0.91, 1.70) | 1.30 (0.92, 1.79) | 0.179 | 1.21 (0.93, 1.71) | 1.26 (0.90, 1.74) | 0.930 |
| LDL-c (mmol/l), mean+SD | 3.25+0.98 | 3.37+1.02 | 0.054 | 3.26+0.99 | 3.26+0.99 | 0.947 |
| HDL-c (mmol/l), mean+SD | 1.64+0.50 | 1.72+0.59 | **0.014** | 1.65+0.52 | 1.69+0.54 | 0.159 |
| Body mass index (kg/m^2^), mean+SD | 26.1+4.5 | 26.5+4.7 | 0.160 | 25.9+4.3 | 26.4+4.8 | 0.088 |
| Systolic BP (mmHg), mean+SD | 131.1+17.0 | 137.4+19.2 | **<0.001** | 131.8+17.8 | 134.4+18.2 | **0.018** |
| Diastolic BP (mmHg), mean+SD | 79.9+9.3 | 82.4+8.6 | **<0.001** | 80.0+9.5 | 81.1+8.6 | 0.052 |
| **Prevalence of CVD risk factor** |  |  |  |  |  |  |
| Elevated total cholesterol, n (%) | 374 (35.1) | 131 (41.9) | 0.090 | 279 (35.5) | 167 (42.1) | 0.036 |
| Hypertension, n (%) | 509 (47.8) | 191 (61.0) | **<0.001** | 382 (48.6) | 211 (53.1) | 0.140 |
| Obesity, n (%) | 171 (17.3) | 65 (21.6) | 0.088 | 122 (16.2) | 75 (19.6) | 0.158 |
| Current smoking, n (%) | 238 (22.6) | 41 (13.3) | **<0.001** | 176 (22.5) | 59 (15.1) | **0.003** |
| Diabetes, n (%) | 49 (5.6) | 16 (6.5) | 0.622 | 37 (5.6) | 25 (7.5) | 0.228 |
| **Rheumatic disease related variables** |  |  |  |  |  |  |
| Disease duration (years), median (IQR) | 7.0 (3.2, 13.7) | 6.6 (3.2, 15.5) | 0.631 | 7.2 (3.6, 14.1) | 6.3 (3.0, 12.9) | 0.099 |
| ESR (mm/h), median (IQR) | 12 (6, 19) | 10 (5, 19) | 0.397 | 12 (7, 20) | 10 (5, 18) | **0.006** |
| CRP (mg/l), median (IQR) | 3 (1, 6) | 3 (1, 6) | 0.528 | 2 (1, 6) | 2 (1, 6) | 0.856 |
| DAS28 (ESR), mean+SD | 2.65+1.25 | 2.73+1.23 | 0.377 | 2.66+1.23 | 2.66+1.23 | 0.891 |
| Remission (<2.6), n (%) | 485 (53.4) | 129 (49.8) | - | 376 (54.4) | 178 (51.9) | - |
| Low disease activity (2.6<3.2), n (%) | 150 (16.5) | 44 (17.0) | - | 114 (16.5) | 51 (14.9) | - |
| Moderate disease activity (3.2<5.1), n (%) | 237 (26.1) | 72 (27.8) | - | 177 (25.6) | 98 (28.6) | - |
| High disease activity (>5.1), n (%) | 36 (4.0) | 14 (5.4) | - | 24 (3.5) | 16 (4.7) | - |
| **Antirheumatic medication, current use** |  |  |  |  |  |  |
| Glucocorticoids, n (%) | 321 (30.1) | 104 (33.2) | 0.294 | 235 (29.9) | 116 (29.2) | 0.820 |
| Methotrexate, n (%) | 600 (56.3) | 145 (46.3) | **0.002** | 445 (56.5) | 207 (52.1) | 0.150 |
| Other sDMARDs, n (%) | 792 (74.3) | 213 (68.1) | **0.029** | 593 (75.3) | 272 (68.5) | **0.012** |
| bDMARDs, n (%) | 495 (46.4) | 105 (33.5) | **<0.001** | 358 (45.5) | 149 (37.5) | **0.009** |

*Patient characteristics and cardiovascular risk factors according to status of anti-citrullinated protein antibody and rheumatoid factor. ACPA: anti-citrullinated protein antibody; RF: rheumatoid factor; SD: standard deviation; LDL-c: low density lipoprotein-cholesterol; HDL-c: high density lipoprotein-cholesterol; BP: blood pressure; ESR: erythrocyte sedimentation rate; CRP: C-reactive protein; DAS28: Disease activity score using 28 joint count; sDMARDs: synthetic disease modifying antirheumatic drugs; bDMARDs: biologic disease modifying antirheumatic drugs.*

**Table S9.** Patient characteristics and cardiovascular risk factors according to current use of biologics

| **Variables** | **IJD (n=3517)** | | **RA (n=1961)** | | **axSpA (n=835)** | | **PsA (n=721)** | |
| --- | --- | --- | --- | --- | --- | --- | --- | --- |
|  | **Users**  **(n=1750)** | **Non-users (n=1767)** | **Users**  **(n=848)** | **Non-users (n=1113)** | **Users**  **(n=518)** | **Non-users (n=317)** | **Users**  **(n=384)** | **Non-users (n=337)** |
| Females, n (%) | 953 (54.5) | 1093 (61.9) | 622 (73.3) | 767 (68.9) | 157 (30.3) | 136 (42.9) | 174 (45.3) | 190 (56.4) |
| Age in years, mean +SD | 53.4+11.3 | 56.8+11.5 | 57.8+11.0 | 60.0+11.3 | 47.3+9.4 | 50.2+9.6 | 51.8+10.2 | 52.2+9.7 |
| Working/student, n (%) | 926 (56.4) | 829 (48.6) | 365 (46.3) | 441 (40.5) | 342 (69.5) | 201 (66.6) | 219 (60.7) | 187 (59.0) |
| Education in years, mean+SD | 13.4+3.3 | 12.4+3.2 | 13.0+3.4 | 12.0+3.2 | 14.0+3.1 | 13.4+3.0 | 13.5+3.0 | 13.0+3.1 |
| **CVD risk factor levels** |  |  |  |  |  |  |  |  |
| Total cholesterol (mmol/l), mean+SD | 5.36+1.08 | 5.38+1.07 | 5.33+1.07 | 5.42+1.10 | 5.32+1.06 | 5.25+1.03 | 5.47+1.11 | 5.34+1.02 |
| Triglycerides (mmol/l), median (IQR) | 1.30  (0.95, 1.88) | 1.29  (0.94, 1.82) | 1.20  (0.90, 1.66) | 1.27  (0.95, 1.80) | 1.32  (0.94, 1.92) | 1.40  (0.90, 1.93) | 1.54  (1.06, 2.35) | 1.29  (0.94, 1.82) |
| LDL-c (mmol/l), mean+SD | 3.24+0.97 | 3.34+0.97 | 3.19+0.97 | 3.32+0.98 | 3.26+0.96 | 3.32+0.96 | 3.32+0.99 | 3.42+0.94 |
| HDL-c (mmol/l), mean+SD | 1.54+0.52 | 1.58+0.50 | 1.65+0.53 | 1.63+0.52 | 1.45+0.48 | 1.49+0.46 | 1.44+0.49 | 1.47+0.43 |
| Body mass index (kg/m^2^), mean+SD | 26.5+4.7 | 26.5+4.5 | 25.9+4.5 | 26.2+4.5 | 26.6+4.5 | 26.0+4.0 | 27.9+4.9 | 28.1+4.9 |
| Systolic BP, mean+SD | 130.6+16.5 | 133.3+17.6 | 130.2+16.2 | 133.7+18.0 | 129.6+16.6 | 130.3+16.9 | 133.1+17.0 | 134.9+16.7 |
| Diastolic BP, mean+SD | 80.8+9.4 | 80.6+9.6 | 79.8+8.9 | 80.1+9.4 | 81.1+9.9 | 80.5+10.5 | 82.5+9.4 | 82.4+9.4 |
| **Prevalence of CVD risk factors** |  |  |  |  |  |  |  |  |
| Elevated total cholesterol, n (%) | 551 (31.5) | 601 (34.0) | 285 (33.6) | 426 (38.3) | 141 (27.2) | 80 (25.2) | 125 (32.6) | 95 (28.2) |
| Hypertension, n (%) | 824 (47.1) | 927 (52.6) | 401 (47.3) | 602 (54.1) | 210 (40.5) | 138 (43.7) | 213 (55.5) | 187 (55.5) |
| Obesity, n (%) | 306 (20.4) | 307 (18.4) | 128 (17.0) | 187 (17.6) | 93 (20.8) | 40 (13.7) | 85 (28.5) | 80 (25.7) |
| Current smoking, n (%) | 319 (18.2) | 388 (22.1) | 162 (19.4) | 257 (23.3) | 95 (18.8) | 67 (21.3) | 62 (16.4) | 64 (19.0) |
| Diabetes, n (%) | 93 (6.4) | 76 (5.6) | 42 (6.0) | 56 (6.1) | 22 (5.2) | 6 (2.8) | 29 (9.0) | 14 (6.0) |
| **Rheumatic disease related variables** |  |  |  |  |  |  |  |  |
| RF+, n (%)  ACPA+, n (%) | -  - | -  - | 358 (70.6)  495 (82.5) | 429 (63.4)  571 (73.3) | -  - | -  - | -  - | -  - |
| HLA-B27+, n (%) | - | - | - | - | 307 (89.0) | 138 (78.9) | - | - |
| Disease duration (years), median (IQR) | 10.5  (5.3, 19.1) | 6.9  (2.6, 13.5) | 10.9  (6.4, 18.4) | 6.6  (2.6, 11.8) | 10.8  (4.1, 21.3) | 9.6  (3.3, 20.6) | 9.0  (4.6, 17.3) | 5.8  (1.7, 11.8) |
| ESR (mm/h), median (IQR) | 8 (4, 17) | 10 (5, 18) | 10 (5, 19) | 11 (6, 19) | 6 (3, 15) | 8 (4, 16) | 7 (4, 15) | 9 (5, 16) |
| CRP (mg/l), median (IQR) | 3 (1, 5) | 3 (1, 6) | 2 (1, 5) | 3 (1, 6) | 3 (1, 5) | 3 (2, 5) | 3 (1, 5) | 3 (2, 5) |
| DAS28 (ESR), mean+SD | - | - | 2.54+1.22 | 2.63+1.26 | - | - | - | - |
| ASDAS (CRP), median (IQR) | - | - | - | - | 1.45  (0.95, 2.44) | 1.91  (1.24, 2.77) | 1.47  (0.96, 2.23) | 1.59  (1.09, 2.54) |
| BASDAI, median (IQR) | - | - | - | - | 0.72  (0.18, 3.63) | 2.03  (0.69, 4.81) | - | - |

*Patient characteristics and cardiovascular risk factors according to current use of biologics. IJD: inflammatory joint diseases, RA: rheumatoid arthritis; axSpA: axial spondyloarthritis; PsA: psoriatic arthritis; SD: standard deviation; LDL-c: low density lipoprotein-cholesterol; HDL-c: high density lipoprotein-cholesterol; BP: blood pressure; RF: rheumatoid factor; ACPA: anti-citrullinated protein antibody; HLA-B27: human leukocyte antigen B27; ESR: erythrocyte sedimentation rate; CRP: C-reactive protein; DAS28: Disease activity score using 28 joint count; ASDAS: Ankylosing spondylitis disease activity score; BASDAI: Bath ankylosing spondylitis disease activity index.*
